# Heartworm Disease (*Dirofilaria immitis*) in Two Roaming Dogs From the Urban Area of Castel Volturno, Southern Italy

**DOI:** 10.3389/fvets.2019.00270

**Published:** 2019-08-28

**Authors:** Mario Santoro, Gianluca Miletti, Lucia Vangone, Luisa Spadari, Stefano Reccia, Giovanna Fusco

**Affiliations:** ^1^Department of Animal Health, Istituto Zooprofilattico Sperimentale del Mezzogiorno, Naples, Italy; ^2^Azienda Sanitaria Locale (ASL), Servizio Veterinario, Caserta, Italy

**Keywords:** heartworm disease, vector borne-disease, filarioid nematode, zoonotic parasite, dirofilariosis

## Abstract

The zoonotic filarioid nematode *Dirofilaria immitis* is transmitted by bloodsucking mosquitoes and causes heartworm disease in dogs and wild canines. In the last decade, *D. immitis* has spread in southern Europe including Italy. Few autochthonous foci of infection have been reported in previously non-endemic areas of southern Italy based only on the identification of microfilariae, antigen and serological tests, and polymerase chain reaction assay from both the blood of dogs and mosquito vectors with no description of cases of heartworm disease in both domestic and wild canines. Here, we report first on two cases of heartworm disease found at post-mortem examination in two roaming dogs from the urban area of Castel Volturno in Campania region of southern Italy. Immunological analyses of 11 roaming dogs from the same pack of those two submitted for necropsy and available necropsy data from the dogs recovered from the Campania region along the past 10 years were both negative for *D. immitis* infection. Although rare in southern Italy, these two cases are noteworthy because *D. immitis* may cause serious human infection. We highlight the need to identify the mosquito vectors of heartworm disease in this area using sensitive molecular assay for *D. immitis* DNA for predicting and controlling the spread of infection. We strongly recommend the control and systematic treatment of the domestic and roaming dogs that could constitute the most important infection reservoir.

## Introduction

*Dirofilaria immitis* (Nematoda: Filariidae) causes heartworm disease, which is widespread throughout tropical and temperate regions of the world including Europe. The disease is transmitted by bloodsucking mosquitoes of the family Culicidae mainly to dogs and wild canines (1, 2); however, several cases of human infection have also been reported throughout the world. In humans, which are not suitable definitive hosts for *D. immitis* infection, their immature forms can reach the pulmonary artery and occasionally the anterior chamber of the eyes, triggering an inflammatory response that may result in pulmonary nodules and blindness, respectively (1–3).

In Italy, the most important area where the disease historically occurs in dogs is the Po river valley in the northern area of the peninsula with prevalence ranging from 22 to 80% (4). Few surveys based on the identification of microfilariae, antigen, and serological tests and polymerase chain reaction assay from both the blood of dogs and mosquito vectors revealed that in the last decade, *D. immitis* spread to the southern regions with the establishment of occasional foci of infection (4–9).

Here, we report for the first time two cases of heartworm disease found at post-mortem examination in two roaming dogs from the Campania region of southern Italy. In addition, immunological analyses of roaming dogs from the same urban area were performed, and available necropsy data from the dogs recovered from the Campania region along the past 10 years also were reviewed to estimate the occurrence and prevalence of *D. immitis* in this geographical area.

## Materials and Methods

**Figure 1:**
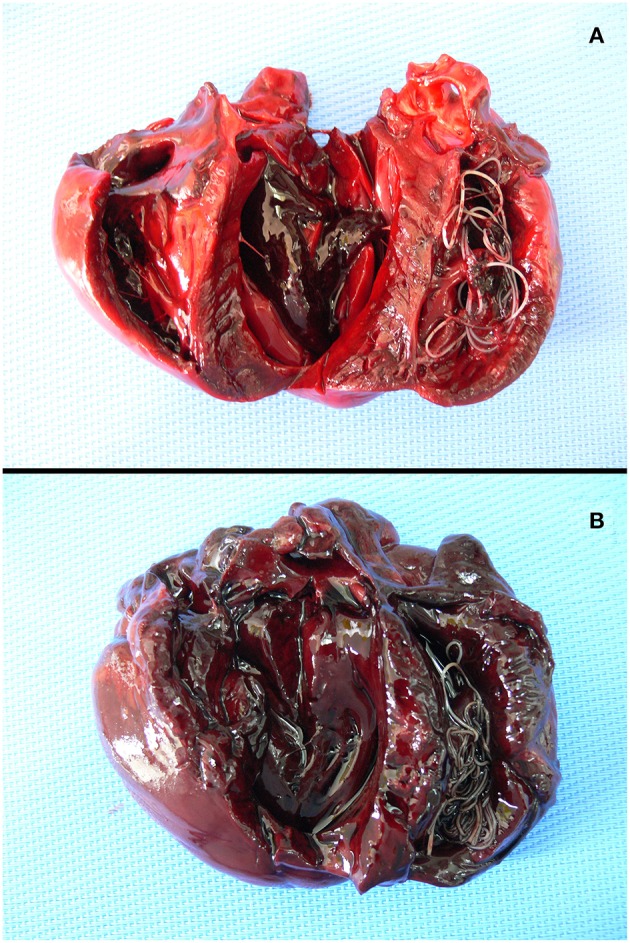
Adult *Dirofilaria immitis* in the heart of male **(A)** and female **(B)** roaming dogs from the urban area of Castel Volturno.

Two fresh carcasses of adult roaming dogs (one male, 2 years old; one female 4/5 years old) with good nutritional status, found on March 5, 2019, in the urban area of Castel Volturno in the Caserta municipality of the Campania region, were submitted to the IZSM (Istituto Zooprofilattico Sperimentale del Mezzogiorno) for post-mortem workup. At routinely parasitological investigation, several large nematodes were observed in the right heart and pulmonary artery of both dogs (Figure 1). Helminths were collected, counted, and preserved in 70% alcohol before examination by light microscopy for morphological identification. Nematode specimens were cleared in lactophenol on a glass slide and identified following Anderson and Bain (10) and Furtado et al. (11).

After the post-mortem examination of the two roaming dogs, in March 2019, blood samples of 11 live roaming adult dogs (including seven females ranging in age from 3 to 6 years old and 4 males ranging in age from 1 to 4 years old) from the same pack and neighborhood of those two submitted for necropsy were collected for immunological analysis. Immunological analyses were performed using an enzyme immunoassay test (The PetChek Canine Heartworm Antigen Test, IDEXX Laboratories Inc.) for the detection of *D. immitis* antigen according to the manufacturer's instructions. The PetChek Canine Heartworm Antigen Test shows a sensitivity of 98% [95% CL (confidence limit) 91.1–100%] and a specificity of 100% (95% CL 99.5–100%).

In addition, retrospective analysis of available necropsy data of dogs recovered from the Campania region (from January 2010 to February 2019) and submitted to the IZSM in Portici were obtained from the database of the IZSM and reviewed to estimate occurrence and prevalence of dirofilariosis during the last decade. Unfortunately, data on the chemoprophylactic treatments used by the dog owners were unavailable.

The IZSM is accredited by the Italian Ministry of Health to perform systematic surveys on infectious diseases of animals. Procedures for this study were performed in accordance with the guide for the care and use of animals by the Italian Ministry of Health.

## Results

**Figure 2:**
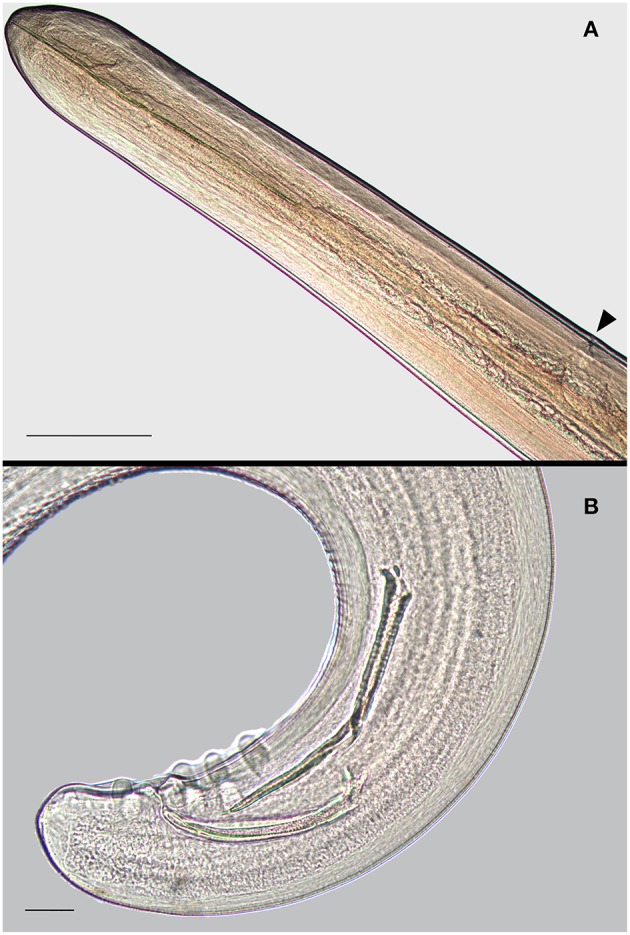
Adult *Dirofilaria immitis*. Anterior extremity of the female **(A)** showing the genital pore (arrowhead); bar = 500 μm. Caudal extremity of the male **(B)** showing the spicules and large ventro-lateral papillae; bar = 50 μm.

From the heart and pulmonary artery of the two roaming dogs, we collected a total of 31 *D. immitis* mature specimens including 11 from the male and 20 from the female (Figures 1, 2). The male/female ratio for *D. immitis* specimens was 1.06:1. Immunological analyses performed on the blood of 11 alive roaming dogs from the same neighborhood of those two submitted for necropsy tested negative for *D. immitis*. Available retrospective necropsy data of dogs submitted to the IZSM in Portici included a total of 1,560 dogs including 729 roaming dogs, and 831 pet dogs. Both classes of those dogs were from all Campania provinces and included 541 dogs from Naples, 383 from Avellino, 274 from Salerno, 168 from Caserta, and 194 from Benevento. No presence of filarioid nematodes from the heart and pulmonary artery was noticed at post-mortem examination by retrospective analyses of necropsy data of those 1,560 dogs.

## Discussion

Our knowledge of *D. immitis* infection in dogs in southern Italy is limited to a few published papers. Cringoli et al. (5), using the modified Knott test and histochemical staining, found microfilariae of *D. immitis* in 2 out 351 dogs (0.6%) from the municipalities of Mt. Vesuvius area in Campania region; however, the two *D. immitis* positive dogs were not native of southern regions but had come from the endemic area of northern Italy. Otranto and Dantas-Torres (6), using both modified Knott test and the commercial Canine Heartworm Test Kit (IDEXX Laboratories Inc.), reported the prevalence of 0.82 and 3.43% in 1,214 and 233 dogs from Apulia and Calabria regions, respectively. Giangaspero et al. (7) from the northern Apulia found that out of 309 privately owned dogs, 11 were positive (3.5%) for at least one *D. immitis* diagnostic test used including the Knott test, the Canine Heartworm Test Kit, and the polymerase chain reaction for *D. immitis* DNA detection. Del Prete et al. (8), using the DiroCHECK ELISA (Synbiotics), found a seroprevalence of 4.4% in 537 kennel dogs from 68 kennels in the Campania region. Unfortunately, no geographical locations were reported for those *D. immitis*-positive dogs. Recently, Piantedosi et al. (9) performed a serological survey in hunting dogs from the Campania region using an in-clinic assay test system (SNAP® 4Dx® Plus, IDEXX Laboratories Inc.), and they found a seroprevalence for *D. immitis* of 0.2% out of 1,335 dogs examined, with the three positive dogs all from the Salerno municipality (9). *D. immitis* reaches sexual maturity in 4 months post-infection, and adults can live over 7 years (2). Considering the known age of at least 2 years old, the time of death of the two dogs, the parasite stage (mature individuals) found in the heart and pulmonary artery, and the mosquito season in southern Italy, we may hypothesize that, at the latest, the infection of the 2-year-old dog occurred in late autumn (October/November). According to Simón et al. (2), the transmission of *Dirofilaria* spp. in a geographical area depends on the presence of dogs infected with adult worms producing microfilariae and on presence of one or more mosquito species suitable to transmit the parasite highlight potential geographical effects in temporal evolution of parasite life stages. Moreover, its transmission is regulated by climate and temperature (below 14°C, the development of L3 larvae inside mosquitoes stops) and may be influenced by human behavior regarding application of chemoprophylaxis and any trips to or from regions of endemicity (2).

Here, we reported on two cases in which adult specimens of *D. immitis* were found in two roaming dogs from the same neighborhood of the urban area of Castel Volturno in southern Italy. We think that both infections were autochthonous. Both dogs were known by the local people and local public veterinary service (ASL); both dogs born and always lived in the neighborhood where they were found dead with no history of travel outside the municipality or history of chemoprophylactic treatments. The site of Castel Volturno is located on the Tyrrhenian coast, and it has a typical Mediterranean climate with long hot summer and mild winters. It has many rural and marshy areas hosting dense populations of susceptible canine reservoirs including feral, roaming, and pet dogs and red fox *Vulpes vulpes* that live in close proximity in the area studied. The area also hosts a large amount of competent mosquito vectors including the most important species (*Aedes albopictus* and *Culex pipiens*) involved in the transmission of *D. immitis* infection in Italy, which, in this temperate area, are feeding at least for 8 months per year (4, 6, 12). Genchi et al. (13) estimated the earliest and latest periods for transmission of *D. immitis* in Naples (about 50 km from Castel Volturno) to be May 11 and October 31, respectively. This large period of transmission is of particular concern in urban areas with a dense canine population that, if the canine reservoirs are infected and not subjected to specific treatment, may provide microfilariae to mosquitos that in turn may infect other canines and humans. No presence of heartworm disease noticed at post-mortem examination by retrospective analyses of those 1,560 dogs from the Campania region confirm that *D. immitis* infection in southern Italy is rare in occurrence. However, it does not confirm that those dogs were negative for larvae and pre-adults of *D. immitis* in their bloodstream since the first preadult worms arrive in the pulmonary artery and right ventricle of canine hearts at between 70 and 85 days post-infection (2). Moreover, the lack of some data (as history of travel and chemoprophylactic treatments) from the retrospective analysis of available necropsies of dogs does not allow the drawing of more conclusive results.

Concerning the immunological negative results of 11 roaming dogs from the same pack of those two submitted for necropsy, Little et al. (14) reported that in some dog populations, a number of samples may be antigen-negative but *D. immitis*-positive. Potential explanations for failing to detect antigen in dogs with *D. immitis* infection may include the following: low worm numbers, male-only infection, presence of immature worms, inappropriate handling or use of an assay, and/or presence of immune complexes (2, 14). Moreover, in dogs, the antigen may not be detectable until 6/9 months post-infection (2, 14, 15). Together with low worm number, it might explain the immunological negative results of the 11 dogs if we consider that from the supposed time of the infection (October/November) of the two necropsied dogs to their death (in March) was at maximum 4/5 months. However, it is also likely that the two positive dogs were the only ones in the pack to be infected by *D. immitis*.

In conclusion, we report the first autochthonous foci of the infection in the urban area of Castel Volturno. Although rare in southern Italy, these two cases are noteworthy because *D. immitis* may cause serious human infection. Based on data presented, studies to identify mosquito vectors using sensitive molecular assay for *D. immitis* DNA (7) in this area are warranted. It could be useful for predicting and controlling the spread of infections; it has been suggested that the geographic expansion and the increased prevalence among canine populations are likely to run in parallel with the increase in numbers of human cases (2). We strongly recommend the control and systematic treatment of the dogs that could constitute the infection reservoir. In addition, due to the zoonotic potential of *D. immitis*, its presence should not be disregarded in humans who come into contact with mosquitoes, which have fed on infected dogs.

## Data Availability

All datasets generated for this study are included in the manuscript/supplementary files.

## Author Contributions

Experimental conception and design were done by MS and GF. Collection of samples was done by GM, LV, and SR. Immunological analyses was done by LS. Analysis, interpretation, and paper writing were done by MS. All authors read and approved the final manuscript.

### Conflict of Interest Statement

The authors declare that the research was conducted in the absence of any commercial or financial relationships that could be construed as a potential conflict of interest.
